# Membrane protein regulators of melanoma pulmonary colonization identified using a CRISPRa screen and spontaneous metastasis assay in mice

**DOI:** 10.1093/g3journal/jkab157

**Published:** 2021-05-08

**Authors:** Louise van der Weyden, Victoria Offord, Gemma Turner, Agnes Swiatkowska, Anneliese O Speak, David J Adams

**Affiliations:** Wellcome Sanger Institute, Cambridge CB10 1SA, UK

**Keywords:** mouse, B16-F0, spontaneous metastasis, lung, melanoma, CRISPR activation, Fut7

## Abstract

Metastasis is the spread of cancer cells to a secondary site within the body, and is the leading cause of death for cancer patients. The lung is a common site of metastasis for many cancer types, including melanoma. Identifying the genes involved in aiding metastasis of melanoma cells to the lungs is critical for the development of better treatments. As the accessibility of cell surface proteins makes them attractive therapeutic targets, we performed a CRISPR activation screen using a library of guide RNAs (gRNAs) targeting the transcription start sites of 2195 membrane protein-encoding genes, to identify genes whose upregulated expression aided pulmonary metastasis. Immunodeficient mice were subcutaneously injected in the flank with murine B16-F0 melanoma cells expressing dCas9 and the membrane protein library gRNAs, and their lungs collected after 14–21 days. Analysis was performed to identify the gRNAs that were enriched in the lungs relative to those present in the cells at the time of administration (day 0). We identified six genes whose increased expression promotes lung metastasis. These genes included several with well-characterized pro-metastatic roles (*Fut7*, *Mgat5*, and *Pcdh7*) that have not previously been linked to melanoma progression, genes linked to tumor progression but that have not previously been described as involved in metastasis (*Olfr322* and *Olfr441*), as well as novel genes (*Tmem116*). Thus, we have identified genes that, when upregulated in melanoma cells, can aid successful metastasis and colonization of the lung, and therefore may represent novel therapeutic targets to inhibit pulmonary metastasis.

## Introduction

Metastasis is the spread of cells from the primary tumor, to a site elsewhere in the body, and is a major cause of morbidity and mortality in melanoma patients. It is a multi-step process in which the primary tumor cells must enter the bloodstream and/or lymphatics (intravasation), survive in the circulation, extravasate into the parenchyma of the target organ(s), and survive in the new environment. Survival and proliferation in this “foreign” microenvironment are necessary for the development of clinically apparent/relevant metastases; as the presence of circulating tumor cells is common in patients with a primary tumor but is not an accurate predictor of metastasis. In many cancers, including melanoma, clinically detectable metastases are primarily found only within a subset of organs, suggesting that interaction of the tumor cell with the specific microenvironment of the secondary tissue is critical. In patients with melanoma, metastasis to the lung is common and often the first clinically detectable site of visceral metastasis (reviewed in Damsky *et al.* 2010). Thus, controlling pulmonary metastasis is of critical importance for patients with melanoma.

Several of the key hallmarks of cancer involve altered expression of membrane proteins and the downstream signaling pathways that they regulate. These have been widely used as biomarkers and therapeutic targets (Lin *et al.* 2019). Membrane proteins expressed on the cell surface represent particularly attractive drug/immunological targets due to their accessibility, such as the receptor tyrosine kinase AXL (which is frequently over-expressed in many cancer types) (Boshuizen *et al.* 2018) and the transmembrane calcium signal inducer TROP-2 (which is highly expressed in metastatic breast cancer) (Rugo *et al.* 2020). Indeed, with critical roles in mediating interactions between the tumor cells and their microenvironment, cell surface proteins are involved in key steps of the metastatic cascade, such as intravasation (Conn *et al.* 2008) and homing to the lung microvasculature (Brown and Ruoslahti, 2004).

CRISPR activation (CRISPRa) screens involve the use of a catalytically inactive version of Cas9 (“dCas9”) fused to transcriptional activator domains (such as VP64), and guide RNAs (gRNAs) that target the transcription start sites (TSSs) of genes. Binding of the dCas9 and gRNA to the TSS of a gene results in its transcriptional activation and thus increased expression of the encoded protein (Horlbeck *et al.* 2016). We previously applied CRISPRa technology to screen a membrane protein gRNA library for genes that enhanced melanoma cell colonization of the lung, in the context of an “experimental metastasis assay” (van der Weyden *et al.* 2021). In the present study, we apply the same gRNA library in an alternative context, specifically the “spontaneous metastasis assay,” which examines the entire metastatic cascade, from growth of the cells in the primary tumor to their spread to a secondary site and eventual colonization of the organ. This screen identified membrane proteins whose upregulated expression in melanoma cells enhanced the ability of the cells to metastasize to the lung and survive and proliferate at that secondary site, and thus represent potential therapeutic targets and/or biomarkers.

## Materials and methods

### Mice

Immunodeficient NOD.Cg*Prkdc^scid^, Il2rg^tm1Wjl^/*SzJ (NSGM) mice were originally obtained from JAX Laboratories and maintained as a core colony at the Sanger Institute Research Support Facility. The care and use of all mice in this study were in accordance with the Home Office guidelines of the UK and procedures were performed under a UK Home Office Project license (P6B8058B0), which was reviewed and approved by the Sanger Institute’s Animal Welfare and Ethical Review Body. All mice were housed in individually ventilated cages in a specific pathogen-free environment. Female NSGM mice aged 6–8 weeks were used in this study, with the diet, cage conditions, and room conditions of the mice as per previously reported (van der Weyden *et al.* 2017).

### Cells

The B16-F0 mouse melanoma cell line was purchased from ATCC (CRL-6475), genetically validated (Del Castillo Velasco-Herrera *et al.* 2018), and maintained in DMEM with 10% (v/v) fetal calf serum (FCS) and 2 mM glutamine, 100 U/mL penicillin/streptomycin at 37°, 5% CO_2_. The cell line was screened for the presence of mycoplasma and mouse pathogens (Charles River Laboratories, USA). B16-F0 cells stably expressing dCas9 (“dCas9-F0 cells”) were generated as described previously (van der Weyden *et al.* 2021).

### CRISPR activation membrane protein screen

The mouse membrane protein (“m6”) mCRISPRa-v2 subpooled library generated by Jonathan Weissman (Horlbeck *et al.* 2016) consists of 10,975 gRNAs targeting the TSSs of 2195 genes that encode for membrane proteins, and 250 nontargeting control gRNAs, and was acquired from Addgene (#84003). The m6 library was re-amplified in liquid culture following Weismann lab protocols (https://weissmanlab.ucsf.edu/CRISPR/CRISPR.html) and the plasmid DNA extracted using an EndoFree Plasmid Mega Kit (Qiagen). The plasmid DNA was packaged into lentiviruses in HEK293T cells using Lipofectamine™ LTX (ThermoFisher) and packaging plasmids pMD2.G (Addgene #12259) and psPAX2 (Addgene #12260). Sixteen hours after transfection, the medium was replaced with complete IMDM and ∼30 hours later, the medium (containing the virus supernatant) was collected and filtered with a 0.45 µm low protein-binding filter (Merck). Transduction was carried out in dCas9-F0 cells at a multiplicity of infection (MOI) of 0.3 and 8 g/mL of polybrene and the medium was changed the following day. Three independent populations of dCas9-F0 cells were transduced and maintained separately (hereafter known as groups A, B, and C). After 48 hours, cells were passaged and 5 μg/mL of puromycin added to the medium. A sample of the transduced cells (and some untransduced cells as controls) were analyzed by flow cytometry (BD LSRFortessa™) to measure the expression of blue fluorescent protein (BFP; a BFP cassette is present in the vector backbone of the “pCRISPRia_v2” library plasmid) and confirm successful transduction. After a further 4 days, cells were passaged again and after 9 days, cells were detached, counted, centrifuged at 300 g for 5 minutes then diluted in phosphate-buffered saline (PBS). Two aliquots of 5.5 × 10^6^ cells from each group (500x library representation) were pelleted and snap-frozen (representing “0-hour” timepoint) and aliquots of 5.5 × 10^5^ cells from each group (50x library representation) in 100 μL PBS were subcutaneously administered into the right flank of 6–8 week old female NSGM mice. Groups A and B had 13 mice each, and group C had 14 mice (making a total of 40 mice). The primary tumor masses on the flank of the mice were measured using Vernier calipers every 1–3 days and the mice humanely sacrificed when the tumor reached ∼2.5 cm^2^ (width of the tumor × length of the tumor).

### Processing the lung samples

The lungs from each mouse (all 5 lobes), as well as the “0-hour” timepoint dCas9-F0 cell pellets, were homogenized in 1 mL Tris-buffered saline with 0.5% Triton X-100 and a representative portion taken for genomic DNA (gDNA) extraction using the Purgene kit (Qiagen) according to manufacturer’s instructions. The gDNA (50 ug) was digested with SbfI HF (NEB) overnight (to enrich for gDNA containing the gRNA as SbfI sites flank the gRNA in the pCRISPRia_v2 vector) and the desired 471 bp product was purified using a Select-a-Size DNA Clean & Concentrator kit (Zymo) according to the manufacturer’s protocol. PCR reactions were performed with 500 ng of the 471 bp product per reaction, using the Phusion^®^ High-Fidelity PCR Master Mix with HF Buffer (NEB) to amplify the gRNAs. The forward primer contained an 8mer barcode, 5’ Illumina adapters and homology to the CRISPRia-v2 plasmid and the reverse primer contained 3’ Illumina adapters and homology to the CRISPRia-v2 plasmid (Horlbeck *et al.* 2016). For each mouse/lung, 24 PCR reactions (of 35 cycles each) were performed and all products were pooled. PCR was also performed on duplicate 0-hour dCas9-F0 cell gDNA. Each set of pooled PCRs was purified using a Monarch^®^ PCR & DNA Cleanup kit (NEB; to purify the 280 bp PCR product), followed by a Select-A-Size DNA Clean & Concentrator kit (Zymo; to remove the 150 bp primer-dimer), according to the manufacturer’s protocol. Purity and concentration of all PCR samples were confirmed by analysis on a Bioanalyser. The samples within each cohort of lungs and relevant “0-hour” timepoint samples (cohorts A–C) were pooled, and each cohort sequenced over 2 runs on a HiSeq2500 (Illumina). Two sequencing primers were used: a bespoke primer (GTG TGT TTT GAG ACT ATA AGT ATC CCT TGG AGA ACC ACC TTG TGG) and a standard Illumina primer.

### Statistics and bioinformatic analysis

Single end 50 bp reads were trimmed to remove adapter sequences and compared for exact matches against the 11,225 sgRNAs (including 250 nontargeting controls) from the Weissman murine membrane proteins “m6” library (which only includes the “Top5” gRNAs) [https://www.addgene.org/pooled-library/weissman-mouse-crispra-v2-subpools/]. Guides with <30 reads in either of the “0-hour” timepoint replicates were removed from the resulting count matrix. To identify enriched gRNAs, the “percentile” method was used as previously described (van der Weyden *et al.* 2021). Briefly, total normalization was performed with MAGeCK (version 0.5.8) (Li *et al.* 2014), using the “0-hour” timepoint samples as the control, and gRNAs with no reads assigned were removed post-normalization. The samples from all 3 cohorts were pooled together for the analysis. For each gRNA, the following was determined: the number of samples (mice) in which it was present (n), the count mean (m), standard deviation (SD), error of the mean (SEM; calculated as SD divided by the square root of the group count), the z-score (determined as the normalized count minus the mean of the control counts, divided by the group SEM) and the number of samples (mice) in which it was present within the 98th percentile (n_NEP; in terms of relative abundance of all gRNA reads in that mouse). gRNAs were ranked by the number of samples (mice) in which they were present in the 98th percentile, followed by z-score.

### Data availability

The data underlying this article are available in the article and its online supplementary material. The CRISPRa data are available under the European Nucleotide Archive (ENA) accession number ERP127223.

## Results

**Figure 1:**
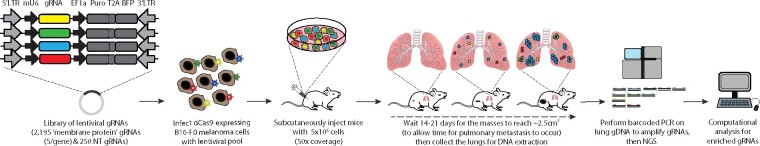
Schematic outline of the screen. dCas9-expressing B16-F10 mouse melanoma cells were transduced with a library of lentiviral gRNAs targeting the TSS of 2195 “membrane protein” genes (five gRNAs/gene) and 250 nontargeting (‘NT’) controls. These cells (5.5 × 10^5^, which represents a 50-fold coverage of the library) were then subcutaneously injected into the flank of immunodeficient mice. The resulting masses were regularly measured and when they reached ∼2.5 cm^2^ (sufficient time for metastasis and pulmonary colonization to have occurred; 14–21 days after administration of the tumor cells), the mice were humanely sacrificed. Their lungs were then collected for genomic DNA (gDNA) extraction and barcoded PCR was used to amplify the gRNAs present in the gDNA. The pooled PCRs were then sequenced using next-generation sequencing (NGS) and bioinformatic analysis was performed to identify the gRNAs that were enriched in each lung/mouse.

To identify membrane proteins whose increased expression enhances the ability of melanoma cells to metastasize to the lung, we performed a spontaneous metastasis assay in mice using the B16-F0 mouse melanoma cell line. In the spontaneous metastasis assay, tumor cells are orthotopically administered to the mouse and form a primary tumor mass over time; some of these cells may then metastasize (enter the circulation, travel to distant sites, and survive and proliferate at that secondary site). It has been shown previously that B16-F0 cells predominantly favor metastasizing to the lung (Deng *et al.* 2017), but they are weakly metastatic (Del Castillo Velasco-Herrera *et al.* 2018), thus, they were chosen to reduce the “background” of the screen (*i.e.*, the number of cells that may metastasize to the lung regardless of the gRNA they are carrying). B16-F0 cells stably expressing dCas9 (F0-dCas9) were virally transduced with a CRISPRa gRNA library targeting genes that encode membrane proteins (Figure 1). The library contained 11,225 gRNAs in total, and was composed of gRNAs targeting 2195 membrane protein-encoding genes (with five gRNAs/gene) and 250 nontargeting “control” gRNAs, cloned into the pCRISPRia_v2 vector backbone (Horlbeck *et al.* 2016). Three populations of F0-dCas9 cells were virally transduced (experiments A, B, and C) and maintained separately throughout the experiment. Mice were subcutaneously administered with 5.5 × 10^5^ cells from either experiment A, B, or C (representing a 50x coverage of the library).

Each experimental cohort was composed of 13–14 immunodeficient (“NSGM”) mice, with 40 mice in total. NSGM mice were used to avoid the issue of ulceration of the tumor mass which occurred in a significant proportion of cases when we used immunocompetent mice (60%, *n* = 36/60 C57BL/6NTac mice, 60%). This can result in death of the mouse due to shock (even in tumors of <1 cm^2^) (Konger *et al.* 2017) and requires euthanasia as per Home Office-approved protocols. Ulceration of subcutaneously administered mouse melanoma B16 cells has been previously reported (van Elsas *et al.* 2001; Erkes *et al.* 2016; Konger *et al.* 2017), and ulceration of melanomas in humans carries a poor prognosis (Tas and Erturk 2021). The developing primary tumor in the flank of the NSGM mice was regularly measured and when it reached ∼2.5cm^2^ (average: 2.33 ± 0.36 cm^2^), which occurred 14–21 days post-dosing (average: 15.2 ± 2.1 days), the mice were humanely sacrificed and their lungs collected for analysis of pulmonary metastases. A “timepoint” of 2.5 cm^2^ was set based on previous experience of the spontaneous metastasis model, as this allowed sufficient time for the cells in the developing mass to undergo pulmonary metastasis and colonize the lung, without allowing the subcutaneous mass to become too large (and risk impeding the locomotion of the mouse). Allowing the mass to continue growing, rather than surgical removal at a small size, avoids the issue of tumor cell seeding during surgery, which may contribute to metastasis formation by the tumor cells that enter the bloodstream (Pachmann 2011). All mice developed a primary tumor that reached the size limit set, however, at this timepoint, the metastases were only macroscopically visible in three of the mice. Details of the individual mice in each cohort, day sacrificed and presence of macroscopically visible lung metastases are shown in Supplementary Table S1.

**Figure 2:**
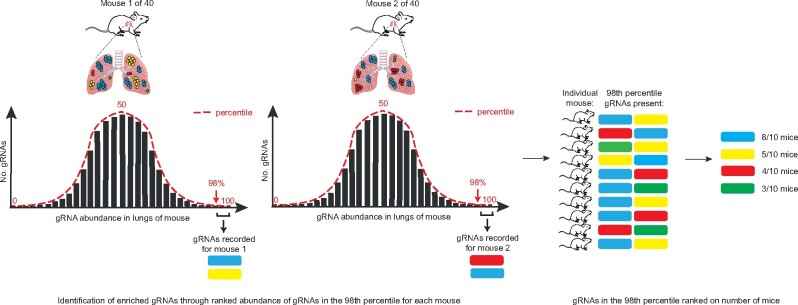
Schematic outline of the percentile ranking approach used to identify enriched gRNAs. To identify gRNAs that were significantly enriched in the lungs of the mice, the gRNAs found in the top 98th percentile of abundance in the lungs of each individual animal was determined. When comparing the percentile ranking results across all 3 cohorts (40 mice total), the gRNAs were then ranked by how often (*i.e.*, in how many mice) they were detected, followed by their z-score.

Genomic DNA (gDNA) was extracted from the lungs of the mice in all 3 cohorts and high-throughput sequencing was performed to identify the gRNA representation in each mouse, relative to that present in the transduced B16-F0-dCas9 cell population before injection (*i.e.*, “0-hour” timepoint). To identify gRNAs that were significantly enriched in the lungs of these mice, we used a “percentile ranking” (PR) approach (van der Weyden *et al.* 2021), to identify gRNAs found in the top 98th percentile of abundance/mouse (Figure 2). This method analyses each gRNA on an individual basis and does not consider the results of the four other gRNAs targeting TSSs of the same gene, as each TSS may not have an equal ability to regulate gene expression. When comparing the percentile ranking results across all 3 cohorts, the gRNAs were ranked by how often they appeared in the top 98th percentile, followed by their z-score (Supplementary Table S2). A biological filter was applied as we were only interested in determining robust (strong) regulators of metastasis and thus, we considered only those gRNAs found in the 98th percentile in >30% of the mice. Using this filter, we classified six gRNAs as hit: *Fut7, Mgat5, Olfr322, Olfr441, Pcdh7*, and *Tmem116* (Table 1 and Supplementary Table S3). The gRNA that had abundance in the 98th percentile in the greatest number of mouse lungs (40%, 16/40) targeted a TSS of *Fut7*. The other five gRNAs had abundance in the 98th percentile in 32.5% of mice (*n* = 13/40).

**Table 1:** Screen hits

Gene	No. mice	z-score
*Fut7*	16/40	10.76
*Olfr441*	13/40	9.04
*Pcdh7*	13/40	8.73
*Mgat5*	13/40	8.54
*Tmem116*	13/40	8.47
*Olfr322*	13/40	6.08

The gRNAs were ranked based on how many times they were found in the top 98% percentile of abundance (considered on a per-mouse basis). Hits were defined as those found in the 98th percentile of >30% of the mice (*n* = 40 mice in total). The number of mice in which the gRNAs were found in the 98th percentile and the z-score for the specific gRNA is shown. Details of the specific gRNAs and the mice in which they were identified are provided in Supplementary Tables S1 and S2.

## Discussion

We previously performed an *in vivo* CRISPRa screen for membrane proteins whose upregulated expression resulted in enhanced metastatic colonization of the lung (van der Weyden *et al.* 2021). This was performed using an experimental metastasis assay in which mice were tail-vein dosed with dCas9-expressing B16-F0 cells transduced with a library of CRISPRa gRNAs targeting the TSS of membrane proteins. We identified several novel genes as playing a key role in pulmonary metastatic colonization, including *Lrrn4cl*, *Slc4a3*, and *Tm4sf19*, and we performed an in-depth characterization of the mechanism of action of *Lrrn4cl* (van der Weyden *et al.* 2021). In the current study, we used the same CRISPRa gRNA library, in the context of a different assay, to further interrogate the role of these membrane proteins in promoting metastasis. Specifically, we performed a “spontaneous metastasis assay,” whereby the tumor cells were subcutaneously administered to the mouse and the metastatic clones arising within the primary tumor spontaneously extravasate into the blood/lymphatic stream. In comparison to the experimental metastasis assay which administers the tumor cells directly into the bloodstream, the spontaneous metastasis assay also includes the early stages of the metastatic cascade, such as the growth of the primary tumor into the surrounding tissue, outgrowth of clones with metastatic potential, and invasion through the basement membrane and into the blood or lymphatic vessels (intravasation). This analysis implicated *Fut7*, *Mgat5*, *Pcdh7*, *Tmem116*, and olfactory receptors (ORs) *Olfr441* and *Olfr322* as drivers of pulmonary metastasis in melanoma cells, with gRNAs targeting the TSSs of these genes being found in the 98th percentile of relative abundance of gRNAs present in the lungs of ≥13/40 mice. Although gRNAs targeting *Lrrn4cl* were found in the 98th percentile in the lungs of 5/40 mice, they were not classified as a “hit” in this screen as we applied a biological filter of having to be found in the lungs of >30% of the mice. In addition, the differences between the assays are likely responsible for the differences in genes identified in the two studies. The experimental metastasis assay interrogates only the terminal stages of the very inefficient metastatic cascade, whereas the spontaneous metastatic assay includes the early steps that happen prior to arrest at the secondary site. Thus, whilst some degree of overlap might be expected in theory since the terminal stages of metastasis are captured in both assays, this requires each screen to have been performed to saturation. However, due to the complex and highly inefficient nature of metastasis, it is unlikely that this depth of analysis would have been achieved. Thus, it is not unexpected to have nonoverlapping gene sets given the numbers of mice (*n* = 35–40), cells (5.5 × 10^5^), and gRNA fold coverage (only 50x) used in both screens.

For the genes implicated as drivers of melanoma pulmonary metastasis that were identified by the spontaneous metastasis assay, some have well-established roles in tumorigenesis and metastasis, whilst the function of others is relatively unknown.

### Fut7

The *fucosyltransferase 7* (*Fut7*) gene encodes a Golgi alpha-(1,3)-fucosyltransferase enzyme that catalyzes the final fucosylation step in the synthesis of the sialyl Lewis X antigen (sLe^X^, also known as CD15s) (Sasaki *et al.* 1994). sLe^X^ can serve as a ligand for cell surface-expressed E-selectin or P-selectin and is involved in selectin-mediated adhesion of cancer cells to the vascular endothelium, a critical step in the metastatic process (Kannagi *et al.* 2004). Expression of *FUT7* is related to poor prognosis in lung cancer (Ogawa *et al.* 1996) and sLe^X^ over-expression is associated with tumor metastasis, recurrence, and overall survival in patients with cancer (Liang *et al.* 2016). FUT7 expression is up-regulated at both mRNA and protein levels in hepatocellular carcinoma HepG2 cells, and an sLe^x^-binding DNA aptamer effectively inhibited the interactions between E-selectin and sLe^x^ in HepG2 cells, resulting in reduced adhesion, migration, and invasion of the cells *in vitro* (Wang *et al.* 2017). *In vitro* studies in A549 lung cancer cell lines have shown that *FUT7* overexpression augments sLe^X^ synthesis to trigger cell proliferation via the activation of the EGFR/AKT/mTOR signaling pathway (Liang *et al.* 2017). Lung cancer and glioma cell lines transfected with *FUT7* cDNA showed increased expression of sLe^X^ and changed their characteristics from “nonmetastatic” to “metastatic”-like (including increased adhesion to a brain-derived endothelial cell monolayer and disruption of an *in vitro* blood-brain-barrier model) (Jassam *et al.* 2019). Thus, elevated *FUT7* expression has a well-established role in metastasis and we show here for the first time that elevated *Fut7* expression can promote the spontaneous metastasis of melanoma cells.

### Mgat5

The *Mgat5* gene encodes the N-acetylglucosaminyltransferase-V enzyme (also known as GnT-V) that produces the β1,6-linked *N*-acetylglucosamine (GlcNAc) branch on the α1,6-mannose residue of *N*-glycans that are attached to cell surface or secreted glycoproteins (Gu *et al.* 1993). *MGAT5* expression levels, which have been shown to be driven by the oncogenic RAS-RAF-ETS1 pathway (Kang *et al.* 1996; Buckhaults *et al.* 1997), are elevated in various cancer types (Zhao *et al.* 2008a, 2008b). Numerous studies have shown that β1,6-branching of glycoproteins promotes tumor progression and metastasis. For example, the presence of β1,6-branched oligosaccharides are increased in lymph node metastases and correlate with poor prognosis in breast carcinoma (Taeda *et al.* 1996) and increased β1-6 branching in nonmetastatic murine mammary carcinoma cell clones strongly correlated with their acquisition of metastatic potential (Dennis *et al.* 1987). The β1-6 branch modification allows target proteins to be covered with a number of *N*-acetyllactosamine (LacNAc) residues, creating high-affinity ligands for galectins (Dennis *et al.* 2009) which can prolong the appearance of growth factor receptors on the cell surface (Boscher *et al.* 2011). The modification also regulates the adhesive properties of the cells by inhibiting cell adhesion and enhancing cell migration (Guo *et al.* 2009), thereby promoting tumor cell invasion and metastasis. Indeed, over-expression of *MGAT5* in pre-malignant lung epithelial Mv1Lu cells resulted in reduced contact inhibition and adhesion, and increased cell motility and transformation (Demetriou *et al.* 1995). Tumor growth and metastasis are strongly suppressed in *Mgat5* knockout mice and cells (Granovsky *et al.* 2000; Guo *et al.* 2010).

#### Olfactory receptors

The *olfactory receptor 322* (*Olfr322*) and *olfactory receptor 441* (*Olfr441*) genes encode ORs which are members of a large family of G-protein coupled receptors involved in the sensory perception of smell. Whilst ORs are mainly distributed in olfactory neurons, they are also expressed in nonolfactory tissues, termed ectopic ORs. Ectopic ORs have been reported to play an important role in tumor progression of many different cancer types (reviewed in Lee *et al.* 2019).

##### Olfr322:

The human homolog of *Olfr322* is *olfactory receptor family 2 subfamily W member 3* (*OR2W3*); the proteins encoded by these genes share 85% amino acid identity. *OR2W3* is one of only 7 ORs whose expression was detected in the chronic myelogenous leukemia K562 cell line and white blood cells from patients with acute myeloid leukemia (Manteniotis *et al.* 2016). Over-expression of OR2W3 has been postulated to play an important role in the development and progression pancreatic cancer, with elevated OR2W3 expression detected in pancreatic cancer tissues by immunohistochemistry (relative to adjacent nontumorous tissue) and the expression of OR2W3 correlating with clinical stage and lymph node metastasis (Shi *et al.* 2018). Similarly, a study investigating the abundance of all 408 human coding OR genes in 960 cases of invasive breast carcinoma and 56 human breast cancer cell lines found significant upregulation of *OR2W3* in a proportion of these tumors; particularly stage IV tumors and those significantly abundant in the expression of genes associated with breast tumor invasion (Masjedi *et al.* 2019). In addition, *OR2W3* upregulation was associated with reduced survival in invasive breast carcinoma, suggesting that it may be a potential breast cancer invasion marker, with possible roles in breast cancer progression and metastasis requiring further investigation (Masjedi *et al.* 2019). Our study would support a role for OR2W3 in metastasis.

##### Olfr441:

The human homolog of *Olfr441* is *olfactory receptor family 2 subfamily A member 14* (*OR2A14*); the proteins encoded by these genes share 79–82% amino acid identity (depending on the transcript). A retroviral insertional mutagenesis screen for mediators of prostate cancer progression, found lung metastases with vector provirus tagging the *OR2A14* gene (3.5 kb downstream of the TSS) (Bii *et al.* 2018). In addition, Oncomine™ analysis of gene expression between prostate patient tissue and unaffected tissue found *OR2A14* was significantly over-expressed (*P* = 0.000145) (Bii *et al.* 2018). Our study adds weight to the role of OR2A14 in cancer progression.

### Pcdh7

The *protocadherin-7* (*Pchd-7*) gene encodes a transmembrane receptor that is a member of the cadherin superfamily. The protocadherin subset of the superfamily have well-established roles in cell adhesion and regulation of downstream signalling pathways (reviewed in Kahr *et al.* 2013). PCDH7 expression is dysregulated in tumorigenesis, with both oncogenic and tumor-suppressive activities reported in a context-dependent manner. For example, decreased *PCDH7* expression has been reported in colorectal (Bujko *et al.* 2015), bladder (Lin *et al.* 2016), and gastric cancer (Chen *et al.* 2017). However, increased *PCDH7* expression has been reported in nonsmall cell lung cancer (Zhou *et al.* 2017) and castration-resistant prostate cancer/neuroendocrine prostate cancer (Shishodia *et al.* 2019). Increased PCDH7 protein expression was observed during tumor progression in prostate cancer tissues and male TRAMP mice (which spontaneously develop prostate tumors following the onset of puberty) (Shishodia *et al.* 2019). Critically, *PCDH7* over-expression in breast and lung cancer cells has been shown to facilitate brain metastasis by promoting the assembly of carcinoma–astrocyte gap junctions composed of connexin 43 (Chen *et al.* 2016). Our findings would support a role for *PCHD7* over-expression in facilitating lung metastasis of melanoma.

### Tmem116

The *transmembrane protein family member 116* (*Tmem116*) gene encodes a type 3a transmembrane protein with a predicted localization mainly in the endoplasmic reticulum (ER) (Wrzesiński *et al.* 2015). The transmembrane (TMEM) family brings together proteins of mostly unknown functions. Studies have shown that TMEM expression can be up- or down-regulated in tumor tissue relative to adjacent normal tissue and there is experimental evidence that TMEMs can function as oncogenes or tumor suppressor genes, with roles in tumor growth/progression, invasion, metastasis, and chemoresistance (though not all pathways have yet been identified) (reviewed in Schmit and Michiels, 2018). Little is known about the function of TMEM116, with only one study to-date reporting a role in cancer; *TMEM116* expression was significantly down-regulated in clear cell renal carcinomas, which correlated with tumor grade, metastasis, and overall survival (Wrzesiński *et al.* 2015). Our findings would suggest the opposite for TMEM116 in melanoma, and as such it may be like TMEM97, which reportedly has decreased expression in some tumor types and increased expression in others (thus showing both tumor suppressive and oncogenic behaviors in a tissue-dependent context) (reviewed in Schmit and Michiels 2018).

## Conclusions

In summary, we have used a spontaneous metastasis assay to screen a library of 2195 membrane protein-encoding genes to identify candidate positive regulators of metastasis in melanoma cell lines. Our screen identified 6 genes whose increased expression promotes the ability of melanoma cells to leave the primary tumor site in the skin, enter the circulation, travel to the lungs, extravasate into the lung parenchyma, and proliferate and successfully colonize the lung. Some of these genes have known roles in metastasis of a range of tumor types to a range of metastatic sites and others represent novel regulators of pulmonary metastasis. The novel candidates we identify will require further follow-up studies to understand their mechanistic contribution to melanoma metastasis, to validate their role in human disease, and to explore their potential as therapeutic targets.

## Supplementary Material

jkab157_Supplementary_Data
