# Supplementary material for: Membrane protein regulators of melanoma pulmonary colonization identified using a CRISPRa screen and spontaneous metastasis assay in mice
Source: G3 (Bethesda). 2021 May 8;11(7):jkab157. doi: 10.1093/g3journal/jkab157 (PMC8495943; doi:10.1093/g3journal/jkab157)
Supplement: jkab157_Supplementary_Data [file jkab157_supplementary_data.zip › jkab157-suppl_data/GENETICS-G3-2021-402466-s01.docx]

**SUPPLEMENTARY MATERIAL**

**Supplementary Table 1**. Details of the mice used in the study. 'Mouse ID' is the name of the mouse. 'Cage ID' is the identification number of the cage in which the mouse was housed for the duration of the experiment. There are the three groups ('Cohorts') in which the mice were dosed: A, B or C. 'Days PD' is the number of days post-dosing (subcutaneous administration of the tumor cells) after which the mouse was humanely sacrificed. 'Lung ID' is the identification number of the lung (all five lobes) that was taken from the mouse for DNA extraction and gRNA analysis. 'Visible lung metastasis' details whether any metastases were macroscopically apparent on the lungs at the time of collection.

**Supplementary Table 2**. Details of the gRNAs identified in the lungs of mice. 'gRNA' (column A) is the ID of the gRNA is as it exists in Weissman's CRISPRa 'm6' sub-library and 'gene' (column B) is gene whose transcription start site is targeted by the gRNA. The values obtained for the cells at day 0 ("d0") are shown in blue (columns C-F) and the values obtained for the lungs at time of collection ("lungs") are shown in green (columns G-J); n = number of samples, m = mean, sd = standard deviation, sem = standard error of the mean. The z-score ("z_lungs_d0"; column K) shown in red, is calculated as detailed in the Materials and Methods. Shown in purple (columns L-N) are the details related to the lungs in which the gRNA was found in the 98th percentile ("high_lungs"). Specifically, the number of lungs in which the gRNA was found in 98th percentile ("sample count"), the IDs of those particular samples ("sample_IDs") and the precentile ranking of the relative abundance that gRNA in those samples ("ranks").
